# The over-the-scope clipping system for treatment of chronic coloenteric fistula: a case report

**DOI:** 10.1186/s12957-015-0628-0

**Published:** 2015-07-17

**Authors:** Gintautas Radziunas, Audrius Dulskas, Oleg Aliosin, Raimundas Lunevicius, Narimantas E. Samalavicius

**Affiliations:** Vilnius University Hospital Santariskiu Clinics, 2 Santariskiu Str, Vilnius, LT–08661 Lithuania; Centre of Oncosurgery, National Cancer Institute, 1 Santariskiu Str, Vilnius, LT–08406 Lithuania; Department of General Surgery, Emergency General Surgery and Major Trauma Centres, Aintree University Hospital NHS Foundation Trust, Lower Lane, Liverpool, L9 7AL UK; Center of Oncosurgery, National Cancer Institute, Clinic of Internal, Family Medicine and Oncology, Faculty of Medicine, Vilnius University, 1 Santariskiu Str, Vilnius, LT–08406 Lithuania

**Keywords:** Over-the-scope clipping, Colorectal cancer, Colorectal fistula, Ovesco

## Abstract

Anastomotic leak in colorectal surgery is not very unusual. The over-the-scope clipping (OTSC) system (Ovesco), which was originally developed to treat intestinal perforation and was tested with animals, might be the choice for the patient. We presented the case of a 63-year-old man with chronic coloenteric fistula. Conservative treatment was unsuccessful. The orifice was then closed with two subsequent clips, and the patient recovered well. To our knowledge, this is the first successful case of coloenteric fistula treatment with Ovesco.

## Background

Anastomotic leakage after a colorectal resection is not very unusual complication (3–21 %), and it has a significant mortality (6–22 %) [1]. The best treatment for the patient is total parenteral nutrition or a diverting ileostomy for 2–3 months with subsequent reversal of ileostomy only when a radiological contrast study shows that the fistula has healed [2].

About 8 years ago, an over-the-scope clip system, called OTSC (Ovesco Endoscopy, Tubingen, Germany), appeared on the market. It was first tested in animal models and the treating of lesions or bleeding of the gastrointestinal tract [3, 4].

Here, we are presenting the case of a male patient, with a chronic coloenteric fistula after colorectal cancer surgery which was successfully treated by endoscopy. To our knowledge, this is the first successful case of coloenteric fistula treatment with Ovesco.

## Case presentation

A 63-year-old male was admitted to our institute complaining of defecation with blood. Cancer in the middle part of rectal ampulla was diagnosed. After examination and multidisciplinary team consultation, neoadjuvant radiotherapy was started.

**Fig. 1:**
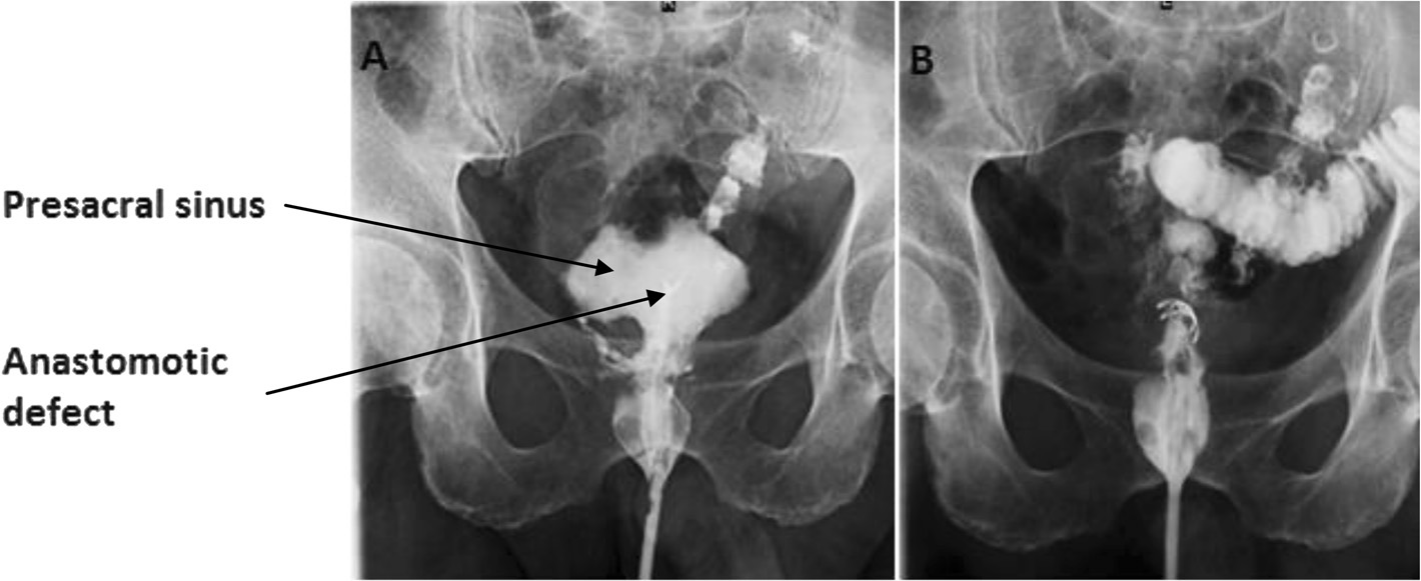
Contrast radiography showing a cave of 10 × 9 × 4 cm and a defect of about 1.5 cm before intervention (**a**, *arrows*) and after applying the OTSC (**b**)

**Fig. 2:**
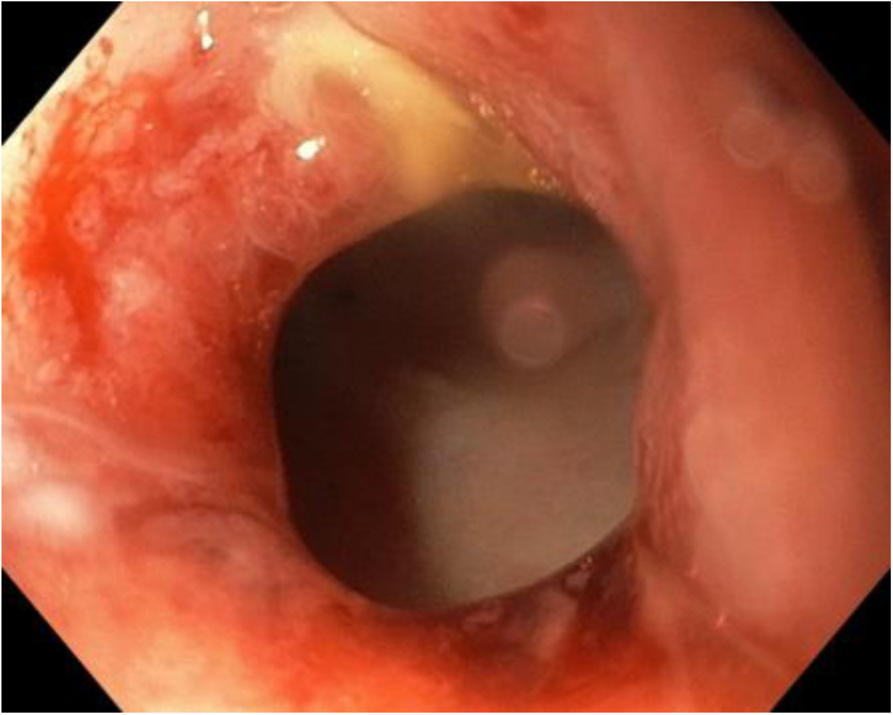
Same anastomotic defect seen on endoscopic evaluation

In 5 days of short-course radiotherapy (25Gy), a low anterior resection with a defunctioning ileostomy was performed. The distance from the anastomosis to the anal verge was 5 cm. The postoperative period was uneventful, and the patient was discharged from the hospital on day 7. On the 9th day after the procedure, the patient was readmitted complaining of urinary retention, and a transcutaneous suprapubic cystostomy was performed. Later, the patient complained of dull pelvic pain, febrile fever, and some liquid discharge through the anus. On a contrast proctography, a presacral sinus of 10 × 9 × 4 cm and a leakage of a short-loop limb after side-to-end anastomosis of about 1.5 cm in diameter were found (Fig. 1a). Since he had a defunctioning ileostomy, conservative therapy was prescribed. After 6 months, an endoscopy was performed. During the procedure, the flexible sigmoidoscope was introduced into the anal canal, and a rectosigmoid anastomosis was seen: an afferent loop of sigmoid colon without abnormalities, in the end of an efferent loop—blind end of the J-shaped colon—a fistula of 1.5 cm in diameter was noted. The flexible endoscope was introduced through the fistula into the cavity (Fig. 2); several openings of the small bowel into the cavity were seen there—during suction, some yellowish, small bowel contents appeared. OTSC system (Over-The-Scope Clip, Ovesco Endoscopy GmbH, Tubingen, Germany) with the clip of t type (blunt teeth, with sharpened ends) was mounted on the endoscope, and the clip was applied on the fistula opening by using suction. Later, urografin (sodium amidotrizoate/meglumine amidotrizoate 30 and 76 %) was injected through the cannula to the site where the clip was applied—the majority of the contrast flew into the colon, but some contrast passed into the cavity. For a week, the patient had no liquid discharge through the anus. Later discharge through the anus appeared again. After 1 month, the procedure was repeated. During the endoscopy, we found that the previously applied clip had almost completely slipped off of the fistula opening, and so, we removed this clip with a polypectomy snare. After removal of the clip, an flexible endoscope was inserted into the cavity, and urografin was injected through the cannula: it was noted that the urografin passed into the small bowel. Another t-type clip was applied on the opening of the fistula using suction, and when we injected a contrast medium after the clip application, no contrast passed through the fistula (Fig. 1b). The patient did very well, and 1 month later, the proctography was normal and the ileostomy was reversed.

## Discussion

The OTSC system is a technique that enables the closure of gastrointestinal defects (perforation sites, leaks, fistulas) and may stop severe bleeding from large lesions of the gastrointestinal tract. For patients who develop a leak after colorectal surgery, treatment can be long and sometimes complicated. A defunctioning ileostomy is not effective in some cases. After the OTSC system application, the patient can be treated at home as was the case with our patient. A successful closure of the leak or fistula is possible when no extraluminal abscess is present [5]. In our case, we had a cavity (previous sinus or abscess) that drained into the small bowel, thereby forming the coloenteric fistula. This allowed us to succeed with a fistula closure, as the cavity could drain into the small bowel.

Looking through the reports, one notes that the success rate of the OTSC system procedure for insufficiency of anastomosis or colorectal fistula was 57–100 % [6], but only nine successful reports of chronic colorectal fistula were found [5, 7–9] (Table 1). A 100 % success rate is reported if the clip is placed within a week of occurrence of the leak [7]. On considering the financial side, clips could reduce costs and time of hospitalization and avoid patients having to undergo a surgical repair [2, 10]. The major advantage of Ovesco clips seems to be their ability to grasp more tissue compared to the standard clips and their strong grip on the wound margins because of their sharpened teeth. The drawback of the clips in fistula sealing is their incomplete grasp when the tissue is fibrotic [2]. Most authors agree that Ovesco is not very appropriate for fistulas larger than 12–15 mm [2, 5].

**Table 1:** Successful reports of chronic colorectal fistula treatment with Ovesco

Author	Number of patients	Success rate (%)	Number of clips
Our case (2015)	1	100	2
Sulz et al. [9]	1	100	1
Arezzo et al. [6]	6	83	6
Manta et al. [10]	2	100	2

## Conclusions

In summary, the application of OTSC appears to be useful in the endoscopic management of colorectal postsurgical fistulas. Further prospective clinical studies are needed to confirm the value and the efficacy of this clipping device.

## Consent

Written informed consent was obtained from the patient for publication of this case report and any accompanying images. A copy of the written consent is available for review by the Editor-in-Chief of this journal.
